# The Gonococcus in Its Relation to Ascending Gonorrhea in Women1Read at the annual meeting of the Medical Society of the County of Erie, January 10, 1893.

**Published:** 1893-03

**Authors:** M. A. Crockett

**Affiliations:** Buffalo, N. Y., Assistant Gynecologist to Buffalo General Hospital, Instructor in Medical Department of the University of Buffalo


### THE GONOCOCCUS IN ITS RELATION TO ASCENDING GONORRHEA IN WOMEN.[1]

By M. A. CROCKETT, M. D., Buffalo, N. Y.,
Assistant Gynecologist to Buffalo General Hospital, Instructor in Medical Department of the University of Buffalo.

Gonorrhea is today classed as an infectious disease, dependent upon a specific microörganism, the gonococcus of Neisser. Since its original discovery in 1879, in the pus of ophthalmia neonatorum, this organism has been studied by many observers, with the result, as expressed by Baumgarten, of placing it "among the best proven microparasitic causes of disease."

In other words, this germ has been cultivated, portions of the pure cultures placed in the human urethra, a typical gonorrhea produced, the pus from this inflammation again yielding pure cultures of the organism. These experiments have been repeated so many times that the specific nature of the gonococcus is as completely established as that of any organism known today. No gonococcus, no gonorrhea.

This organism is a diplococcus, in form resembling the two halves of the coffee berry. It is usually found in groups of ten to twenty individuals, surrounded by an atmosphere of mucus, or situated in the interior of pus or epithelial cells. In an acute case the cocci are readily found. A drop of pus dried, stained with gentian violet or methyl blue, mounted in balsam and examined with oil emersion, will present these organisms in large numbers. Unfortunately, not less than five pseudo-gonococci have been described, which fact renders necessary other tests before positive conclusions can be drawn.

Wherever the primary infection may be, there is no question but that in women the cervix is the favorite habitat of the gonococci, and that from this region it is most difficult to dislodge them. Frequently lying hidden away between folds of mucous membrane, or in the mouths of glands they are ever on the lookout for fresh fields to conquer. This is the state of affairs in "latent gonorrhea," as described by Noeggerath. Acute manifestations may have passed or never been present; nevertheless, deep down in the crypts of the cervix, a few individuals are clinging tenaciously to life, weak and enfeebled though they may be. Plant these gonococci in fresh territory, where more favorable conditions for their existence can

1. Read at the annual meeting of the Medical Society of the County of Erie, January 10, 1893.

be found, and they awake like giants refreshed and produce an acute inflammation as an evidence of their presence. In women, pregnancy, the puerperal period, menstrual epoch, or any accidental congestion of the genital organs may offer an opportunity for further developments of these latent organisms. Too frequently the disturbance resulting in such cases is mistaken for fresh infection from without, whereas it is merely an exacerbation of a disease already present. Or again, without evidences of acute gonorrhea, these organisms of attenuated strength may gradually, under favoring conditions, creep into the uterus, and from there into the tubes, and produce no symptoms beyond those of a chronic salpingitis. Such a course commonly results in sterility, because the abdominal end of the tube becomes closed, and this affords an explanation of the fact why so many prostitutes are sterile. Another result ascribed to the presence of the gonococci is tubal pregnancy. The cilia of the cells being destroyed before the fimbriated extremity has become closed, the ovum remains in the tube instead of being carried to the uterus.

Very recently studies regarding the action of gonococci in the peritoneum have been made. There have been a number of deaths from general peritonitis, resulting from gonorrheal infection, but in these cases it is not certain that the gonococci have been the sole cause of the result. This question has not yet been definitely settled. There is no doubt, however, but that the gonococcus, alone and unaided, can produce a circumscribed inflammation of the peritoneum and play a very important part in the production of adhesions, always present in severe tubal inflammation. The main point I wish to impress is that the gonococci reach the tubes step by step, and never in leaps, and that during their progress there are points where they can be destroyed and their further advance prevented.

There is another question closely allied with that of gonorrheal infection, and that is the question of mixed infection. Mixed infection is defined as—

The invasion of the organism by two or more kinds of bacteria, there being a causal connection between the invasion of the two germs. In such cases the bacterium possessing the greatest power of invasion precedes and, as it were, prepares the soil for the second germ. These forms of mixed infection are distinguished by a certain constancy of occurrence, and the possibility, and even probability, exists that if one germ has settled another will follow. Ordinary croupous pneumonia, with its after diseases, is a typical example. The bacteria of pneumonia deprive the alveoli of the lungs of their epithelium, and fill them with an exudation which is a soil in which secondarily tubercle bacilli or pyogenic germs settle, and which may lead to the termination of the illness in phthisis, or formation of abscesses.—*Sinclair*.

This theory of mixed infection has been applied by Bumm to the results of invasion by the gonococcus. It is claimed, for instance, that a previous gonorrhea renders a puerperal patient easily subject to a secondary invasion of pyogenic bacteria, giving rise to the so-called gonorrheal puerperal fever. In the tubes, also, it is claimed, the gonococci affect the tissues merely superficially, the formation of pus with destruction of tissue being due to a secondary infection by other germs.

This theory of mixed infection, ingenious as it is, is not necessary to explain the production of pyosalpinx after gonorrheal infection. I fail to find any instance recorded where the gonococcus has been found associated with any other bacterium in the tubes. It seems to me this condition of association must be found before we can accept the mixed infection theory as proven. On the other hand, Wertheim has reported seven cases where in the pus of pyosalpinx he found active gonococci, but *no other* bacteria. Furthermore, he found these gonococci not limited to the subepithelial tissue, but penetrating the wall of the tube and extending into the peritoneum itself. His investigations were most carefully made, pure cultures of the gonococcus obtained from the pus, and the male urethra inoculated in order to draw positive conclusions. The idea that the gonococcus alone cannot produce pus seems to be erroneous, for in these cases no other pyogenic bacterium was present in the tube.

We cannot, however, conclude that mixed infection never plays a part in gonorrheal pyosalpinx. It may afford an explanation of the behavior of some cases of pyosalpinx. Every operator knows that even in comparatively recent cases he may find a large collection of pus in the tubes with little or no general disturbance. On the other hand, some gonorrheal cases of pyosalpinx give rise to symptoms from the first. There is a tendency for the pus to escape from the tube, and there are a number of cases on record where the pus has either discharged through some of the hollow viscera or into the abdominal cavity, producing a fatal peritonitis. Such cases are the result of mixed infection, the pus containing the common, virulent pyogenic bacteria, and showing the same tendency to make its escape by destruction of tissue as is displayed by acute abscess in other parts of the body.

The quiescent cases of pyosalpinx are probably due to the gonococcus alone, an organism limited in its action and comparatively short-lived. In several cases of pyosalpinx of but a few months' standing the pus has been found to be absolutely sterile, the local and general disturbance being very slight, the gonococci no longer finding sufficient nutrition in the tissues. This explains why escape of such pus during operation is followed by no bad results. In an operation reported by me a few months ago this escape of pus occurred to a greater extent than I have ever seen before or since; the intestines were literally floating in pus, and for twenty-four hours pus discharged through the drainage tube showing that some remained behind after closure of the abdomen. Had this pus not been sterile, certainly my patient would not have made the rapid recovery she did. To sum up, then, we may say (*a*) gonococci are the cause of gonorrhea. (*b*) They may remain latent for a long time in the cervix. (*c*) In the tubes and elsewhere they may produce purulent inflammation. (*d*) In some cases other germs are associated—the so-called mixed infection. (*e*) Gonorrheal pyosalpinx may result from simple or mixed infection. (*f*) The behavior of the pus in the tubes varies widely in the two cases.

*Mode of Infection.*—Gonorrhea in women usually results from an impure coitus, the gonococci being deposited in the vagina or thrown directly into the cervix. There are other possible modes of infection, however. In virgins and children the disease has occurred without sexual connection. Epidemics of the disease have been reported where every member of a family or school has become infected. Discharge from a specific ophthalmia or urethritis may be conveyed by the fingers, instruments, towels, clothing, etc. Even the water-closet, that time-honored excuse, is not without its dangers, and under certain circumstances can be a medium of infection. The great danger which constantly menaces the female is from the male patient who has been pronounced cured of his gonorrhea. Acute manifestations of urethritis in the male are obvious enough; the danger lurks in the chronic or gleety stage of the disease. There are several reasons why so many wives become infected from a chronic gleet in their husbands. First, because the doctor, not appreciating the risks to which a future wife may be exposed, does not impress upon the man the importance of continuing treatment until completely cured. Secondly, the doctor doesn't

*know* when the case is cured, and frequently discharges his patient too soon. Thirdly, the man himself, suffering little or no discomfort and not knowing the possible disastrous results, abandons his physician and later marries.

There seems to me to be a wide missionary field open to the general practitioner, namely, the instruction of the laity. Very few men would knowingly inflict upon a woman all the misery of gonorrheal salpingitis. Let it be impressed upon mankind in the most forcible language what a horrible disease gonorrhea may become in a woman, and we shall have both the physician and patient coöperating to diminish the dangers. Every general practitioner must awake to the fact that women's lives are being ruined every day, for which he is often responsible. Through him alone can the laity be instructed, but bear in mind that the blind cannot lead the blind.

How can we tell when a case of chronic gonorrhea is completely cured and all danger of infection removed? By bearing in mind that as long as there are gonococci present in the discharge, the case is dangerous. In chronic cases these bacteria are few and of attenuated strength, but when transposed to the healthy genital tract of the female they may increase sufficiently to give rise to an acute inflammation, or, on the other hand, may produce the latent and creeping form of the disease, giving no manifestations of their presence until the tubes have become involved. It takes about five minutes to prepare a drop of a suspected secretion for the microscope. In the acute or subacute forms of inflammation there is usually no difficulty in detecting the gonococci when present. Unfortunately, in a chronic gleet, on the other hand, it is extremely difficult to find the specific microörganism, owing to their paucity of numbers or admixture with other bacteria. The only sure method of detecting the gonococci in such cases is by making cultures. Until recently it was very troublesome to cultivate this germ, but Wertheim has perfected the technique so that any pathologist with a fairly-equipped laboratory can readily test a suspected discharge. By Wertheim's method the gonococci were detected every time, in spite of their small numbers and accidental or artificial mixture with other bacteria. In a chronic case, the urethra should be stimulated by the passage of a sound or injection of nitrate of silver solution and a little of the discharge resulting be used for cultures. This entails trouble, to be sure, but suppose your patient wishes an opinion as to whether he can marry safely or not? Knowing what you do about this disease in women, can you conscientiously neglect any means of informing yourself about the condition of this man's urethra? Let me close this paper with a clinical picture. Last week I had in my office a patient with the following history: At the age of nineteen she was a bright, intelligent girl, having an occupation which not only enabled her to support herself, but through which she had accumulated over two hundred dollars in a savings bank. About six months ago she married. Within six weeks she had an attack of pelvic inflammation which confined her to her bed for several weeks. She got up a wreck. She has now a retroverted, bound-down uterus, enlarged tubes, and a vaginal vault so sensitive that she can hardly bear a cotton tampon in the vagina. The diagnosis was plain enough and was confirmed by her asking me one day whether I thought an inflammation her husband had before marriage was in any way responsible for her present condition. It seems that her husband was treated for gonorrhea several months before marriage. He was discharged cured, and he particularly inquired whether he could safely marry. He married in good faith and is completely broken down to see what he has brought upon his wife. The wife's savings are already spent, and by lying down two days she with difficulty works one, and is dragging out a miserable existence. Later, probably, I shall be obliged to perform a laparatomy.

Every gynecologist meets numbers of such cases and sees where the blame should rest. Sänger has said that the time would come when a father would inquire more closely into the condition of a prospective son-in-law's urethra and less into the condition of his pocket-book. When that time comes, there will be a falling off in the number of abdominal sections.

